# Oxidative Stress & Male Infertility - A necessary and conflicted indissociable marriage: How and when to call for evaluation?

**DOI:** 10.1590/S1677-5538.IBJU.2019.0751.1

**Published:** 2021-01-20

**Authors:** Jorge Hallak, Thiago A. Teixeira

**Affiliations:** 1 Laboratório de Andrologia Clínica e de Pesquisa de Alta Complexidade Centro de Ciência e Inovação São PauloSP Brasil Androscience - Centro de Ciência e Inovação em Andrologia e Laboratório de Andrologia Clínica e de Pesquisa de Alta Complexidade, São Paulo, SP, Brasil.; 2 Universidade de São Paulo Divisão de Urologia São PauloSP Brasil Divisão de Urologia, Universidade de São Paulo – USP, São Paulo, SP, Brasil.; 3 Universidade de São Paulo Institute for Advanced Studies Men's Health Study Group São PauloSP Brasil Men's Health Study Group, Institute for Advanced Studies, Universidade de São Paulo – USP, São Paulo, SP, Brasil.; 4 Universidade de São Paulo Unidade de Toxicologia Reprodutiva São PauloSP Brasil Unidade de Toxicologia Reprodutiva, Universidade de São Paulo – USP, São Paulo, SP, Brasil.; 5 Universidade Federal do Amapá Faculdade de Medicina MacapáAP Brasil Faculdade de Medicina da Universidade Federal do Amapá - UNIFAP, Macapá, AP, Brasil.

*To the editor*,

The biological formation of hydrogen peroxide has been postulated since 1922, particularly in connection with catalase and peroxidase's presumed function, initially only found in cultures of some bacteria and some molds and specific enzymatic reactions requiring molecular oxygen for the oxidation of their respective substrates (1-5). In 1943, indirect evidence for H_2_O_2_ formation during respiration of bovine spermatozoa in the egg-yolk medium had been demonstrated, and that the incubation of human spermatozoa under conditions of high oxygen tension resulted in a loss of motility that could be reversed by the presence of catalase (6,7). In 1946, finally, hydrogen peroxide formation by human spermatozoa and its inhibitory effect on respiration was demonstrated (8). In 1986, the positive association between impaired sperm function and men attending infertility clinics was established (9). In 1989, Aitken et al. described that human spermatozoa can generate reactive oxygen species (ROS), significantly accelerated in cases of defective sperm function, examining the pivotal role of lipid peroxidation (LPO) in mediating free radical damage to cells, either by an intrinsic mechanism or induced by ionophore (9). Among functional consequences, was a dose-dependent reduction in human spermatozoa's ability to exhibit sperm-oocyte fusion, which could be reversed by including a chain-breaking antioxidant, alfa-tocopherol, implicating a causative role for LPO in the etiology of defective sperm function. Furthermore, a possible physiological role for ROS generated by human spermatozoa in mediating physiological spermatozoa-oocyte interaction was postulated (9).

The biological failure of reproduction and the consequential involuntary childlessness experienced by an increasing population of males worldwide expressing a 59.7% decrease in semen quality over less than 40 years in 43 thousand men in 85 studies from all continents, should raise concern to a potential common, unique and powerful sperm cell damage mechanism: oxidative stress (10). The generation of reactive oxygen and nitrogen species, leading to peroxidation of lipids in the bi-lipidic sperm membrane, ultimately fragmenting the mitochondrial and nuclear DNA and damaging other organelles.

Decades later, although these concepts appear simple and straightforward, the pathophysiological understanding, the laboratorial and clinical applications of ROS & LPO measurements for male component infertility have not yet been widely disseminated outside the inner circle of translational andrology. In spite that a recent Medline search using the terms spermatozoa and oxidative stress/ROS/LPO found 2,006 papers, the andrological community linked to male subfertility is still struggling to disseminate to urologists, reproductive endocrinologists, and gynecologists that regular semen analysis is insufficient and imprecise to translate functional status at a cellular level. The purposeful and convenient misinterpretation by many of what means low-quality or abnormal seminal parameters has unfortunately led to a growing, profitable, relatively uncontrolled, and under sighted multi-billion dollar, industry leading to excessive use and abuse of assisted reproductive technologies in many parts of the world (11), with consequences going beyond the couple´s health but diving deep into future generations faced with unknown potential risks that include, but are not limited to: higher miscarriage rates, congenital disabilities, premature or low-birth-weight, multiple gestations, higher maternal death, increased and major urogenital, cardiovascular and neurological malfomations, increased genomic instability, increased imprinting disorders, De novo mutations, increased risk of hypospadias, higher blood pressure at a young age, increased risk of cancer, psychological and neurodevelopmental features in offspring after ART treatments (11-16). The purpose of a high-complex and fully dedicated andrology laboratory with internal and external quality control assessments in the settings of andrology/urology practices is mandatory to investigate the underlying cause of infertility thoroughly and at the same time guide to improving methods to restore male general health, testicular and sperm function. Andrologists should assess sperm cell status through selective functional tests and ultimately propose methods to enhance this evaluation, searching for reliable, reproducible, cost-effective tests with well-established reference values for the fertile and infertile population.

Over time, field-tested methodologies have been defined as the gold-standard OS and ROS measurement method, the chemiluminescence assay using a luminometer and luminol (5-amino-2,3-dihydro 1,4-phthalazinedione) as a probe. Results are expressed as 10^4^ counted photons per minute (cpm)/ 20 × 10^6^ sperm. Each andrology setting could establish its own local/regional reference values for the fertile population following strong World Health recommendations. Athayde et al., 2007 in Brazil established reference values for ROS in neat and washed semen samples with or without leukocytes for the population with proven fertility (16). For washed semen, it was established 51.5 as an optimal cutoff (accuracy 73.2%) for samples with leukocytes but without Leukocytospermia, and 10.0 for semen without leukocytes (accuracy 69.0%). For neat semen, an optimal cutoff of 0.56 for samples without leukocytes and 1.25 for semen with leukocytes (Endtz >0 and <1 × 10^6^/mL) was determined (17). Of notice, the identification of potential sources of ROS is essential for guiding any clinical intervention. Leukocytes are a significant ROS production source (18) and solidly indicate the demand for a more careful evaluation. A significant difference was identified when comparing the fertile groups with and without leukocytes (excluding samples with Leukocytospermia). Even fertile men had substantially higher ROS levels in neat and washed semen samples with leukocytes (<1 × 10^6^/mL).

Nevertheless, one point to be considered is that ROS are just a part of the spectrum of oxidative stress that can be precisely measured by various and laborious techniques, other by-products like reductive nitrogen species, sometimes even more harmful, fall short in the capacity of testing. Functionally speaking, sperm capacitation is expressed as a redox-regulated mechanism wherein a low level of reactive oxygen species (ROS) generation is closely involved in driving such events as promoting the cAMP generation, cholesterol efflux reinforcement, and tyrosine phosphorylation. However, ROS's continuous generation ultimately creates problems for spermatozoa because their unique physical architecture and unusual biochemical composition mean that they are vulnerable to oxidative stress. Therefore, spermatozoa and other testicular cells are heavily dependent on the antioxidant protection afforded by the fluids in the male and female reproductive tracts and, including the seminal plasma. Suppose this antioxidant protection should be compromised for any reason. In that case, the spermatozoa experience pathological oxidative damage, to such a degree that spermatozoa under normal physiological conditions or even to some extent and for a period, pathological circumstances (ex.: varicocele, cigarette smoking, marijuana use), spermatozoa can engage in a redox-regulated cascade of hyperactivation, capacitation, and even sperm-zona pellucida binding, without fear of succumbing to OS, but with a thin margin (18).

Cicek et al. (19) aimed at measuring the association of seminal oxidation-reduction potential (ORP) with sperm parameters in patients with unexplained male infertility. A “one-size-fits-all” system is uniquely seductive in the positive sense that by analyzing all components in the spectrum of OS and the counterpart of antioxidants, the expressed number would reflect a positive or negative biological homeostasis that could guide the clinician towards treatment antioxidants or other medical interventions. Several questions are raised and should be of concern. Although it is attractive to have a specific number that expresses the fight between OS and defense mechanisms, the measurement of all these elements and their by-products made by the human spermatozoa and surrounding tissues is made particularly difficult because of the various oxygen metabolites involved and the low levels of ROS generation compared with contaminating cell types, in particular neutrophils. Therefore, any study that does not adequately address these variables should be looked at with precaution. The reduction of one single electron that provides enough energy to generate input for the chemiluminescence or any other electrical stimulation assay, could also be achieved by reductases such as cytochrome b5 and P450 using NADH or NADPH as electron donors, respectively. This could lead to a serious bias or flaw in methods to measure OS or ORP, provided that no positive or negative controls are running at the same time. Furthermore, as confounding-factors not addressed in the paper by Cicek et al. are the potential presence of defective immature spermatozoa with excessive residual retained cytoplasm, quite common in the infertile population, which by itself can generate excessive OS via NAD(P)H oxidase (20).

The ORP measurement could theoretically have some advantages as it arguably provides redox balance in real-time and can be assessed even in frozen seminal plasma, requiring less technical expertise than other techniques (21). But what about quality control issues and calibration bias? Nonetheless, criticisms also emerge because the sample's viscosity directly interferes with results, not to mention other interferences like neutrophils and other OS generating cells. To be widely accepted, more independently evaluated prospective studies are mandatory with larger sample sizes, fertile and infertile controls, and more widely standardized reference values for all these variables with or without Leukocytes. Anyway, the good news is that Andrology is moving progressively forward with concepts that sooner or later will become easily more understandable.

## References

[1] 1. McLeod JW, Gordon J. Production of Hydrogen Peroxide by Bacteria. Biochem J. 1922;16:499-506. 10.1042/bj0160499 PMC1259100 16743107

[2] 2. Neill JM, Avery OT. Studies on oxidation and reduction by pneumococcus : v. The destruction of oxyhemoglobin by sterile extracts of pneumococcus. J Exp Med. 1924;39:757-75. 10.1084/jem.39.5.757 PMC2128526 19868882

[3] 3. Sevag MG, Maiweg L. The respiration mechanism of pneumococcus. III. J Exp Med. 1934;60:95-105. 10.1084/jem.60.1.95 PMC2132380 19870289

[4] 4. Pearce AA. On the so-called “iodide oxidase”. Mechanism of iodide oxidation by Aspergillus. Biochem J. 1940;34:1493-500. 10.1042/bj0341493 PMC1265437 16747280

[5] 5. Coulthard CE, Michaelis R, Short WF, Sykes G. Notatin: an anti-bacterial glucose-aerodehydrogenase from Penicillium notatum Westling and Penicillium resticulosum sp. nov. Biochem J. 1945;39:24-36. 10.1042/bj0390024 PMC1258144 16747849

[6] 6. Tosic J, Walton A. Respiration of spermatozoa in egg-yolk medium. Nature 1945; 156: 507-8.

[7] 7. MacLeod J. The role of oxygen in the metabolism and motility of human spermatozoa. American Journal of Physiology 1943; 138: 0512-8.

[8] 8. Tosic J, Walton A. Formation of hydrogen peroxide by spermatozoa and its inhibitory effect of respiration. Nature. 1946;158:485. 10.1038/158485a0 20999112

[9] 9. Aitken RJ, Clarkson JS, Fishel S. Generation of reactive oxygen species, lipid peroxidation, and human sperm function. Biol Reprod. 1989;41:183-97. 10.1095/biolreprod41.1.183 2553141

[10] 10. Levine H, Jørgensen N, Martino-Andrade A, Mendiola J, Weksler-Derri D, Mindlis I, et at. Temporal trends in sperm count: a systematic review and meta-regression analysis. Hum Reprod Update. 2017;23:646-59. 10.1093/humupd/dmx022 PMC6455044 28981654

[11] 11. Hallak J. A call for more responsible use of Assisted Reproductive Technologies (ARTs) in male infertility: the hidden consequences of abuse, lack of andrological investigation and inaction. Transl Androl Urol. 2017;6:997-1004. 10.21037/tau.2017.08.03 PMC5673814 29184804

[12] 12. Meister TA, Rimoldi SF, Soria R, von Arx R, Messerli FH, Sartori C, et at. Association of Assisted Reproductive Technologies With Arterial Hypertension During Adolescence. J Am Coll Cardiol. 2018;72:1267-74. 10.1016/j.jacc.2018.06.060 30190005

[13] 13. Kahraman S, Sahin Y, Yelke H, Kumtepe Y, Tufekci MA, Yapan CC, et at. High rates of aneuploidy, mosaicism and abnormal morphokinetic development in cases with low sperm concentration. J Assist Reprod Genet. 2020;37:629-40. 10.1007/s10815-019-01673-w PMC7125256 31901112

[14] 14. Halvaei I, Litzky J, Esfandiari N. Advanced paternal age: effects on sperm parameters, assisted reproduction outcomes and offspring health. Reprod Biol Endocrinol. 2020;18:110. 10.1186/s12958-020-00668-y PMC7664076 33183337

[15] 15. Aitken RJ, De Iuliis GN, Nixon B. The Sins of Our Forefathers: Paternal Impacts on De Novo Mutation Rate and Development. Annu Rev Genet. 2020;54:1-24. 10.1146/annurev-genet-112618-043617 32663048

[16] 16. Esteves SC, Roque M, Bedoschi G, Haahr T, Humaidan P. Intracytoplasmic sperm injection for male infertility and consequences for offspring. Nat Rev Urol. 2018;15:535-62. 10.1038/s41585-018-0051-8 29967387

[17] 17. Athayde KS, Cocuzza M, Agarwal A, Krajcir N, Lucon AM, Srougi M, et at. Development of normal reference values for seminal reactive oxygen species and their correlation with leukocytes and semen parameters in a fertile population. J Androl. 2007;28:613-20. 10.2164/jandrol.106.001966 17409462

[18] 18. Lemkecher T, Dartigues S, Vaysse J, Kulski O, Barraud-Lange V, Gattegno L, et at. Leucospermie, stress oxydatif et fertilité masculine: certitudes et hypothèses [Leucocytospermia, oxidative stress and male fertility: facts and hypotheses]. Gynecol Obstet Fertil. 2005;33:2-10. French. 10.1016/j.gyobfe.2005.01.001 15752659

[19] 19. Cicek OSY, Kaya G, Alyuruk B, Doger E, Girisen T, Filiz S. The association of seminal oxidation reduction potential with sperm parameters in patients with unexplained and male factor ınfertility. Int Braz J Urol. 2021;47:112-9. 10.1590/S1677-5538.IBJU.2019.0751 PMC7712687 33047916

[20] 20. Gomez E, Buckingham DW, Brindle J, Lanzafame F, Irvine DS, Aitken RJ. Development of an image analysis system to monitor the retention of residual cytoplasm by human spermatozoa: correlation with biochemical markers of the cytoplasmic space, oxidative stress, and sperm function. J Androl. 1996;17:276-87. 8792218

[21] 21. Agarwal A, Roychoudhury S, Bjugstad KB, Cho CL. Oxidation-reduction potential of semen: what is its role in the treatment of male infertility? Ther Adv Urol. 2016;8:302-18. 10.1177/1756287216652779 PMC5004233 27695529

